# DSP-0509, a TLR7 agonist, exerted synergistic anti-tumor immunity combined with various immune therapies through modulating diverse immune cells in cancer microenvironment

**DOI:** 10.3389/fonc.2024.1410373

**Published:** 2024-09-13

**Authors:** Yosuke Ota, Ryosaku Inagaki, Kentaro Sumida, Megumi Nakamura, Yasuhiro Nagai, Setsuko Yamamoto

**Affiliations:** Cancer Research Unit, Sumitomo Pharma Co., Ltd., Osaka, Japan

**Keywords:** TLR7 agonist, DSP-0509, myeloid cell, IDO1, epacadostat, AXL, TP-0903, single-cell RNA sequencing

## Abstract

Toll-like receptor 7 (TLR7) acts as a crucial component of the innate immune system. Upon TLR7 binding to its ligand, myeloid cells, including dendritic cells (DCs) and macrophages, are activated and play vital roles in initiating adaptive immunity. Consequently, TLR7 agonists have been employed in cancer immunotherapy. We have synthesized DSP-0509, a systemic injectable TLR7 agonist, and in this investigation, we examined the effects of DSP-0509 on tumor-infiltrating lymphocytes (TILs) utilizing single-cell RNA sequencing (scRNA-seq) in a mouse model bearing tumors. Our results demonstrated that DSP-0509 induced an expansion of immune cell populations, such as Natural Killer (NK) cells, CD4^+^ T cells, and CD4^+^ regulatory T cells (Tregs). Subsequently, we combined an Indoleamine 2,3-dioxygenase 1 (IDO1) inhibitor with DSP-0509 to enhance the antitumor efficacy by reducing Tregs, as DSP-0509 led to an increase in Treg presence within tumors. Our findings demonstrated that this combination therapy effectively reduced Treg infiltration within the tumor, leading to enhanced antitumor activity. To further prevent CD8^+^ T cell exhaustion, we combined DSP-0509 with an anti-PD-1 antibody and assessed the alterations in TILs using scRNA-seq. Our results indicated that the combination treatment significantly increased the cluster of CD8^+^ T cells expressing Gzmb, Prf1, Ctla4, and Icos, when compared to the administration of DSP-0509 alone. Additionally, we observed a marked rise in the M1-like macrophage cluster in the combination treatment group compared to the group receiving only DSP-0509. To validate the potential of modulating myeloid cells within the tumor to enhance antitumor efficacy, we combined DSP-0509 with an inhibitor targeting the receptor tyrosine kinase AXL. In bone marrow derived macrophages (BMDMs), the AXL inhibitor further amplified DSP-0509-stimulated TNFα secretion while reducing IL-10 secretion. As a final step, we evaluated the antitumor activity by combining DSP-0509 and the AXL inhibitor in an *in vivo* tumor model, which demonstrated increased efficacy. In summary, our study elucidated the effects of DSP-0509 on immune activity within the tumor microenvironment. These findings provided valuable insights that pave the way for the development of novel combination immunotherapy strategies.

## Introduction

Because they have demonstrated the capacity to extend the effectiveness of therapy, cancer immunotherapies, such as anti-PD-1 antibodies, have revolutionized and superseded the standard-of-care across numerous cancer types (1). However, despite the efficacy of immunotherapy, only a subset of patients derive benefits, leaving ample room for improvement. Therefore, numerous studies aim to enhance the efficacy in non-responsive patients by exploring combined immunotherapy as an avenue of investigation (2). Specifically, the combination of drugs targeting multiple stages in the cancer immune cycle is anticipated to elicit robust antitumor immune responses in patients who do not respond to monotherapy (3). An exemplification of this approach is the utilization of both anti-LAG3 antibody and anti-PD-1 antibody (4, 5). Toll-like receptors (TLRs) play pivotal roles in the immune defense against viruses and bacteria (6). As pattern-recognition receptors, TLRs identify pathogen-associated molecular patterns (PAMPs) (viruses- and bacteria-specific structures and nucleic acids), as well as damage-associated molecular patterns (DAMPs) that emerge during cellular death (6). TLR7 predominantly participates in the initiation of viral immunity. Specifically, TLR7 binds to viral single-stranded RNAs and activates innate immunity via the MYD88 pathway (7). TLR7 is broadly expressed in myeloid cells and exhibits strong expression in plasmacytoid dendritic cells (pDCs) (8). *In vivo*, pDCs are recognized as the principal source of type I interferon and function as tumor cell eliminators (8). Consequently, TLR7 agonists have garnered attention as promising candidates for anti-tumor immunotherapies. However, the approval of imiquimod is limited to localized administration only (9), necessitating the development of systemically available TLR7 agonists to tackle tumors that are not superficially located (10). Previously, we have demonstrated that DSP-0509, a systemically available TLR7 agonist, exhibits potent antitumor effects when combined with anti-PD-1 antibody, concomitant with the presence of memory T cells (11). Nevertheless, given that TLR7 is expressed in myeloid cells that exert early influence on the anti-tumor immune response and affect various immune cell populations, it is expected that DSP-0509 would prove effective not only in conjunction with anti-PD-1 antibodies but also in combination with other immunotherapeutic agents. AXL is a member of the TYRO3, AXL, MER (TAM) family of receptor tyrosine kinases. It has been reported that TAM family members contribute to the proliferation, invasion, and epithelial-to-mesenchymal transition of cancer cells. Furthermore, AXL is implicated in the attenuation of immune responses in myeloid cells (12). Studies indicate that TLR signaling triggers activation of the AXL pathway, which, in turn, suppresses TLR signaling as a negative feedback mechanism to terminate the immune response (13, 14). Considering the evidence of TAM family involvement in cancer progression, clinical development of inhibitors targeting TAM members, including AXL, is currently underway as potential anti-tumor therapeutics (12). IDO1 catalyzes the conversion of tryptophan to kynurenine, thereby mediating immunosuppression and playing a pivotal role in the pathogenesis of autoimmune diseases and the differentiation of Tregs (15). IDO1 is expressed on both cancer cells and myeloid cells within the tumor microenvironment, where it elevates the level of kynurenine, an immunosuppressive metabolite, while depleting tryptophan, a vital nutrient for effector cells, including cytotoxic T lymphocytes (CTLs) (16). Additionally, IDO1 expression in DCs has been shown to promote Treg differentiation (16). It is worth noting that induction of IDO1 expression in response to interferon-gamma (IFNγ), an effector molecule of CTLs in the tumor, is recognized as one of the tumor’s mechanisms of evading immune surveillance (17). Consequently, a diverse array of IDO1 inhibitors are presently under development as potential anti-tumor immunotherapies (18). In this study, we employed scRNA-seq analysis to examine the impacts of DSP-0509, either alone or in combination with anti-PD-1, on the immune cell composition in the tumor microenvironment. Furthermore, we demonstrate that the combination of anti-tumor immune agents with DSP-0509 enhances its efficacy, as evidenced by alterations in the immune cell profile.

## Materials and methods

### Compounds and dosing solutions

DSP-0509 was chemically synthesized at Sumitomo Pharma, Co., Ltd. For *in vitro* studies, 10 mM solutions were prepared by dissolution in DMSO followed by dilution of the DMSO up to a final concentration of 0.1%. For *in vivo* studies, DSP-0509 was dissolved in 2.5 mM glycine buffered solution of pH 10.2. Epacadostat was chemically synthesized at Sumitomo Pharma Co., Ltd. For *in vivo* studies, the compound was dissolved in saline containing 3% N, N-dimethylformamide and 10% 2-hydroxypropyl-beta-cyclodextrin. TP-0903 tartaric acid was chemically synthesized at Tolero Pharmaceuticals, Inc. For *in vivo* studies, the compound was dissolved in distilled water containing 5% D-α-tocopheryl polyethylene glycol succinate and 1% Tween 80.

### Cells and cultures

CT26 and 4T1 cells were obtained from the American Type Culture Collection. Renca cells were kindly provided by Dr. Fujioka, Iwate Medical University School of Medicine. HM-1 cells were obtained from RIKEN BRC. The CT26, 4T1, and Renca cells were maintained in culture by passage 1 or 2 times a week in RPMI1640 supplemented with 10% FBS and penicillin/streptomycin. HM-1 cells were maintained in culture by passage 1 or 2 times a week in MEMα supplemented with 10% FBS, and penicillin/streptomycin.

### Mice

We used 6- to 10-week-old female Balb/c mice purchased from The Jackson Laboratory Japan, Inc. or CLEA Japan, Inc., and 6- to 10-week-old female B6C3F1 mice purchased from The Jackson Laboratory Japan, Inc. All animal studies were conducted in compliance with the Sumitomo Pharma Animal Ethics Code.

### *In vivo* anti-tumor study

CT26, 4T1, Renca, and HM-1 cells were suspended in HBSS. CT26 cells were subcutaneously implanted into Balb/c mice. 4T1 cells were implanted into the mammary fat pads of Balb/c mice. HM-1 cells were subcutaneously implanted into B6C3F1 mice. The number of CT26 and 4T1 implanted cells was 1 × 10^6^. For HM-1 and Renca tumor implantation, the number of cells was 1 × 10^5^. When the tumors reached approximately 100 mm^3^, the mice were randomly divided into groups. Bolus i.v. DSP-0509 was administered at 1 or 5 mg/kg and each dose was administered once a week until the end of the study. Anti-PD-1 antibody (Bio X Cell Inc., RMP1-14) was administered twice weekly at 200 μg intraperitoneally in all studies. TP-0903 30 mg/kg was orally administered in a 5-days on/2-days off schedule. Epacadostat 100 mg/kg was orally administered once a day. The tumor volume was calculated using the formula (L × W^2^)/2, where L and W refer to the length and width dimensions, respectively.

### Flow cytometry analysis

Tumors excised from mice immediately after euthanasia were used for isolation of tumor-infiltrating lymphocytes (TILs). After cutting the tissue into 2-3 mm^3^ squares, single cells were acquired from the pieces using a mouse tumor dissociation kit (Miltenyi Biotec B.V. & Co. KG) and gentleMACS™ Octo Dissociator (Miltenyi Biotec B.V. & Co. KG). Additionally, TILs were isolated by treatment with ammonium-chloride-potassium lysing buffer (ACK) buffer to remove the erythrocytes after density-gradient centrifugation using Percoll (GE Healthcare). Flow cytometry analysis was carried out using the following primary antibodies: PE-CD11b (BD Biosciences, M1/70), FITC-Gr-1 (BD Biosciences, RB6-8C5), FITC-CD8a (BD 53-6.7), FITC-CD4 (eBioscience, Inc., RM4-5), PE-CD25 (eBioscience, Inc., PC61.5), APC-CD45RB (eBioscience, Inc., FJK-16s), PE-Cy7-CD11b (BD Biosciences, M1/70), and PE-CD86 (BD Pharmingen, GL1), and the following viability dyes: Fixable Viability Stain 450 (BD Horizon) and Fixable Near-IR Dead Cell Stain (Invitrogen). Foxp3/Transcription Factor Staining Buffer kit (eBioscience, Inc.) or Treg Detection kit (Miltenyi Biotec B.V. & Co. KG) was used for intracellular staining. The flow cytometry data acquisition and analysis of TILs were conducted on a MACSQuant Analyzer 10 Flow Cytometer (Miltenyi Biotec B.V. & Co. KG) using Flow Jo (BD Biosciences).

### scRNA-seq

CT26 tumors were dissociated into single cells using a gentleMACS™ Octo Dissociator and a mouse Tumor Dissociation Kit (Miltenyi Biotec B.V. & Co. KG). CD45^+^ cells were isolated using a FACS Aria™ Fusion cell sorter (BD Biosciences) after presorting with anti-mouse CD45 MicroBeads (Miltenyi Biotec B.V. & Co. KG). The cDNA library was prepared from cells using a Chromium Controller and Chromium Next GEM Single Cell 3′ GEM, Library & Gel Bead Kit v3.1 (10x Genomics, Pleasanton, CA). The cDNA library was sequenced using a NovaSeq 6000 sequencer (Illumina, Inc.). Mapping of the sequence data to the genome and the counting of reads per feature were performed using CellRanger software (ver. 6.1.2, 10x Genomics). Count data were analyzed in the R environment (ver 4.1.0) mainly using the Seurat package (ver. 4.9.9). Initial normalization and scaling were performed with default settings. Clustering of cells was performed using the FindNeighbors function with the top 10 principal components, and using the FindClusters function with a resolution of 0.5 (for whole cells and CD8^+^T cells fraction) or 0.4 (for the monocyte-macrophage-cDC fraction). In order to annotate cell types, whole cells were firstly clustered and gene expression levels of cell type markers were manually examined to clearly identify lymphocytes, the monocyte-macrophage-cDC fraction, pDCs, granulocytes, and mast cells. Similarly, lymphocytes and the monocyte-macrophage-cDC population were separated into T cells and NK cells, and into monocytes, macrophages, and cDCs, respectively, based on clustering and manual examination of markers. T cells were further separated into CD8^+^ or CD4^+^ T cells based on non-zero expression of Cd8a or Cd8b1 gene and zero expression of Cd4 gene for CD8^+^T cells and *vice versa* for CD4^+^T cells. Differentially expressed genes in a specific cluster compared to other clusters in CD8^+^T cells or macrophages were selected using the FindMarkers function with parameters: test.use = “wilcox”, logfc.threshold = 0, and min.pct = 0.2.

### Treg suppression assay

The Treg suppression assay was performed as described previously (19). In brief, Tregs (CD4^+^CD25^+^CD45RB^low^) and CD4^+^T cells (CD4^+^ CD25^-^ CD45RB^high^) from the spleens of CT26 tumor-bearing mice were separated by a MoFlo Astrios cell sorter (Beckman Coulter, Inc.) after pre-sorting with mouse CD90.2 MACS beads (Miltenyi Biotec B.V. & Co. KG). CD4^+^T cells were co-cultured with Tregs and CD90.2-splenocytes stimulated with anti-CD3 antibody (0.1 μg/mL) in presence or absence of DSP-0509 (100 nM) at the indicated ratios for 3 days. CD4^+^T proliferation was evaluated from the rate of decrease in CellTrace^™^ Violet(CTV)-positive fraction. Proliferation index was calculated using FlowJo™ 10.3 (BD Biosciences).

### Treg differentiation assay

CD4^+^T cells and CD11c^+^DCs were isolated from spleens derived from Balb/c mice using anti-CD4^+^T cell and anti-CD11c^+^DC microbeads, respectively (Miltenyi Biotec B.V. & Co. KG). CD4^+^T cells and CD11c^+^DCs were co-cultured at a 2:1 ratio in culture medium containing IL-2 (250 U/mL) and TGFβ (5 ng/mL) in anti-CD3 antibody-coated plates for 4 days in the presence or absence of DSP-0509 (1 μM). The extent of Treg differentiation was analyzed using a Treg detection kit (Miltenyi Biotec B.V. & Co. KG).

### Bone marrow derived macrophage assay

Bone marrow was isolated from Balb/c mice. Bone marrow cells were collected by flushing bone marrow out of bones with HBSS. Bone marrow cells were seeded in 96-well plates, 2 x 10^4^ cells/well, and cultured in the presence of M-CSF (10 ng/mL) for 6 days to induce their differentiation into BMDMs. BMDMs were cultured in the presence of TP-0903 for 1 day, then DSP-0509 was added for 1 day before collection of culture supernatant. The concentrations of mouse IL-10 and TNFα in supernatants were quantified by ELISA (R&D Systems, Inc.).

### Bone marrow derived DC assay

Bone marrow was isolated from Balb/c mice. Bone marrow cells were collected by flushing bone marrow out of bones with HBSS. Bone marrow cells were cultured with GM-CSF (10 ng/mL) for 9 days. The resulting BMDCs were cultured in the presence of DSP-0509 or TP-0903 or both for 2 days. CD86^+^ expression on CD11c^+^ cells was used as an index of DC activation.

### Myeloid derived suppressor cell suppression assay

Monocytic MDSCs (mMDSCs) isolated from tumors derived from Renca tumor-bearing mice were sorted by a FACS Aria (BD Biosciences) cell sorter and identified as the CD11b^+^Gr-1^mid^ fraction. CD8^+^T and CD4^+^T cells were isolated from splenocytes of Balb/c mice by using an EasySep Mouse CD8 or CD4 T-cell isolation kit (STEMCELL Technologies Inc.). Isolated CD8^+^T or CD4^+^T cells were co-cultured with mMDSCs (1:1) for 3 days in the presence or absence of Dynabeads Mouse T-activator CD3/CD28 (Thermo Scientific) and DSP-0509. The concentration of IFNγ in the supernatant was measured by using a mouse IFNγ ELISA Kit (R&D Systems Inc.).

### Macrophage cytotoxicity assay

Tumor-associated macrophages were isolated from CT26-Her2 tumors in mice treated with or without DSP-0509 5 mg/kg i.v. on day 1 and day 7. The tumors were collected at day 11 and cut into 2-3 mm^3^ pieces. Single cells were prepared from the pieces using a mouse tumor dissociation kit (Miltenyi Biotec B.V. & Co. KG) and gentleMACS™ Octo Dissociator (Miltenyi Biotec B.V. & Co. KG). F4/80^+^ macrophages were isolated by using anti-F4/80 microbeads (Miltenyi Biotec B.V. & Co. KG) with an autoMACS Pro Separator (Miltenyi Biotec B.V. & Co. KG). CT26-Her2 cells were co-cultured with isolated macrophages for 5 h at the indicated ratio. Cytotoxicity for the tumor was detected by the CytoTox 96 Non-radioactive Cytotoxicity Assay (Promega Corporation).

### Statistical analysis and reproducibility

Stat Preclinica Client (SAS 9.4, Takumi Information Technology Inc.) was used for statistical analysis in the *in vivo* anti-tumor studies. The unpaired two-tailed *t*-test was used to compare the data between two groups. In *in vivo* tumor studies, the parametric Dunnett test or Tukey test was used for *post hoc* analysis. Data are presented as mean ± SEM. Regarding scRNA-seq single-cell data, the difference in frequencies of cell types or clusters between the vehicle and specific dosing groups was evaluated by the Fisher exact test with Benjamini-Hochberg (B-H) adjustment for multiple comparison. Differentially expressed genes were identified by the Wilcoxon test with B-H adjustment. For *in vitro* study, reproducibility was confirmed at least 3 independent experiments. For *in vivo* experiment, reproducibility was confirmed by using at least 4 mice.

## Results

### scRNA-seq of TILs revealed that DSP-0509 induced the infiltration of a wide variety of immune cells in tumors

**Figure 1:**
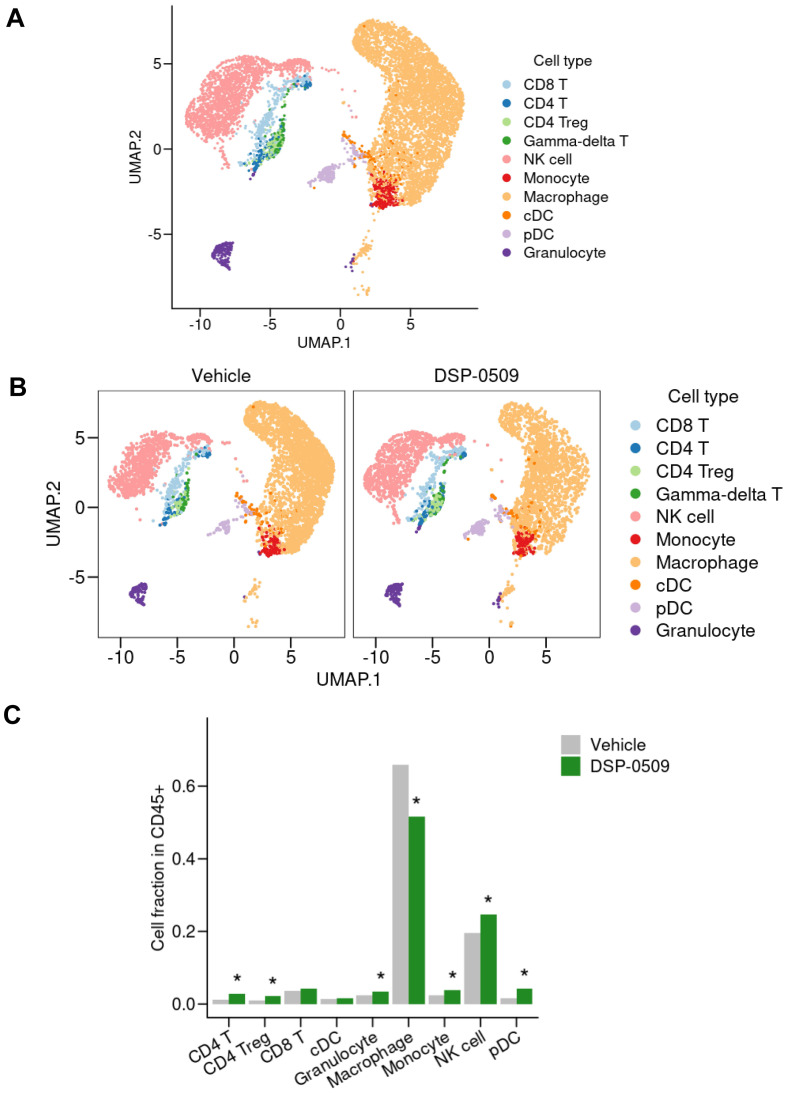
scRNA-seq of TILs revealed that DSP-0509 induced the infiltration of a wide variety of immune cells in tumors. **(A, B)** Uniform manifold approximation and projection (UMAP) representation of cell populations found by the clustering of CD45^+^ cells derived from CT26 bearing mouse from all groups **(A)** or each group **(B)**. Tumor infiltrated CD45^+^ cells were isolated from the CT26-bearing mouse 4 days after administration of vehicle or DSP-0509 5 mg/kg intravenously. CD45^+^ cells in each group were pooled from 2 mice. Isolated CD45^+^ cells were used for scRNA-seq analysis. **(C)** The quantification of the cell fraction of **(B)**. *Adjusted P < 0.05 in the Fisher test.

We previously demonstrated that systemic administration of DSP-0509 exhibits anti-tumor efficacy by stimulating CD8^+^ T cell activation and infiltration in mouse models with tumors (11). However, considering the presence of a diverse array of immune cells within the tumor microenvironment, it is plausible that DSP-0509’s effects extend beyond CD8^+^ T cells to encompass other immune cell populations. Therefore, using a CT26 tumor-bearing mouse model, we conducted scRNA-seq analysis to investigate the impact of DSP-0509 on various immune cell populations within the tumor microenvironment. Tumors were harvested four days after administering DSP-0509 at a dosage of 5 mg/kg. CD45^+^ enriched TILs were isolated by cell sorting after enzymatic digestion of the tumor mass, resulting in a single-cell suspension. Approximately 5,000 cells from both the vehicle and DSP-0509 treatment groups underwent scRNA-seq analysis, yielding approximately 3,000 detectable genes per cell. Based on the similarity of gene expression profiles, cells from both groups were segregated into distinct clusters, with each cluster annotated according to its corresponding immune cell type (**Figure 1A**). The most prevalent cells identified were F4/80-positive cells, primarily macrophages. NK cells, characterized by the expression of CD94, were the subsequent most abundant cell population. Clusters of T cells were also observed, including CD8^+^ T cells, CD4^+^ T cells, and, within the CD4^+^ T cell cluster, a subgroup of Tregs, marked by the expression of Foxp3 (**Figure 1A**). The frequency of cells belonging to each cluster varied between the vehicle and DSP-0509 treatment groups (**Figure 1B**). Notably, we observed a significant increase in the percentages of CD4^+^ T cells, Tregs, NK cells, monocytes, pDCs, and granulocytes in the DSP-0509 treatment group compared to the vehicle group. Conversely, the percentage of macrophages was significantly decreased in the DSP-0509 treatment group (**Figure 1C**). These findings indicated that DSP-0509 activated diverse immune cell populations through TLR7, leading to anti-tumor immune activity via modulation of the immune cell landscape within the tumor microenvironment.

### DSP-0509 modulated the activities of myeloid cells including macrophages and MDSCs

**Figure 2:**
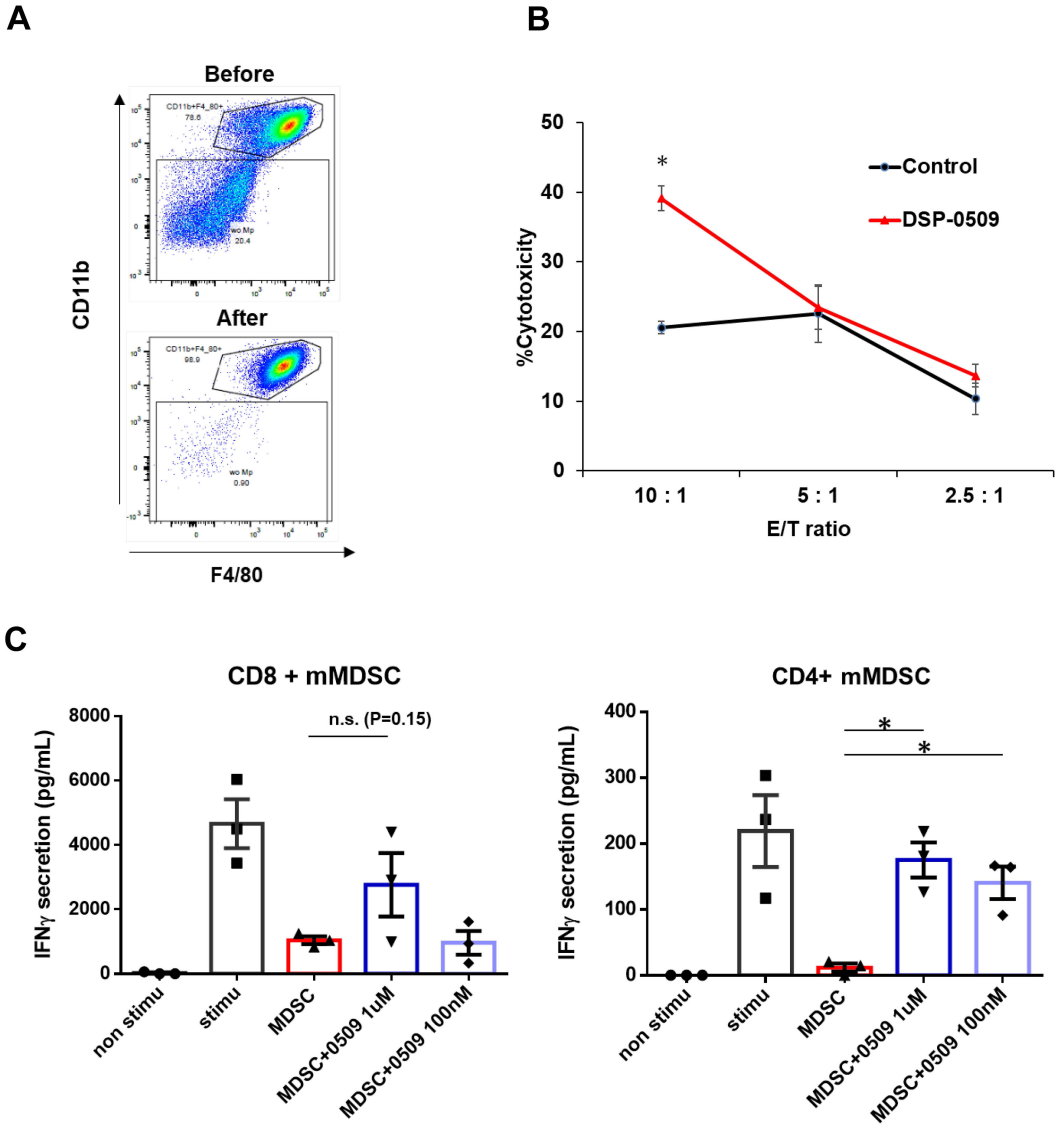
DSP-0509 modulated the activities of myeloid cells including macrophages and MDSCs. **(A)** Representative dot plot of the tumor associated macrophage population before and after MACS microbeads isolation. Tumor associated macrophages were defined as the CD11b^+^F4/80^+^ fraction. **(B)** Macrophages were isolated from CT26-Her2 tumors in mice treated with or without DSP-0509 5 mg/kg i.v. once weekly. The tumors were collected at day 11, and F4/80^+^ macrophages were isolated. CT26-Her2 cells were co-cultured with macrophages for 5 h at the indicated ratio. *P < 0.001 for the Student *t* test. Values are the mean ± S.E.M. of each group, n = 3/group. **(C)** mMDSCs were isolated from Renca tumor-bearing mice by using a cell sorter as the CD11b^+^Gr-1^mid^ cell fraction. Isolated CD8^+^ or CD4^+^ T cells were co-cultured with mMDSCs (1:1) for 3 days. Values are the average ± S.E.M. n = 3/group. *P < 0.001 in the parametric Dunnett test vs the MDSC group. n.s., not significant.

We have clarified that DSP-0509 decreased macrophage in Tumor (**Figure 1C**). To confirm the impact of these alterations on the anti-tumor functionality of TAMs, we evaluated the tumoricidal activity of TAMs in an ex vivo system. Specifically, we isolated TILs consisting of CD11b^+^ F4/80^+^ cells obtained from CT26-Her2 tumor-bearing mice that either received no treatment or were treated with DSP-0509 (**Figure 2A**). Following co-culturing with cancer cells at various ratios for 5 hours, we assessed the tumoricidal activity of TAMs by quantifying lactate dehydrogenase (LDH) concentration in the supernatant. Encouragingly, the tumoricidal activity exhibited a proportional increase in an effector (E) to target (T) ratio-dependent manner (**Figure 2B**). Furthermore, a comparison between the control and DSP-0509 groups revealed a notable augmentation in tumoricidal activity by TAMs in the presence of DSP-0509, particularly at an E/T ratio of 10:1 (**Figure 2B**). Previous studies have reported the suppressive effect of TLR7 agonists on myeloid-derived suppressor cells (MDSCs), which are recognized as immunosuppressive cell populations (20, 21). Consequently, we explored the influence of DSP-0509 on the immunosuppressive functionality of MDSCs. We isolated TILs (CD11b^+^Gr-1^mid^ cells) from Renca tumor-bearing mice and utilized them as monocytic MDSCs (mMDSCs), in addition to isolating CD8^+^T and CD4^+^T cells from the spleen of the same animals. Subsequently, mMDSCs were co-cultured with either CD8^+^T cells or CD4^+^T cells at a 1:1 ratio, in the presence of anti-CD3/CD28 stimulation, over a period of 3 days. The activation of T cells was evaluated by measuring interferon-gamma (IFNγ) concentration in the culture supernatant. Consequently, the secretion of IFNγ increased significantly in CD8^+^T cells and CD4^+^T cells upon anti-CD3/CD28 stimulation. Conversely, the addition of mMDSCs resulted in a reduction of IFNγ levels. However, when DSP-0509 was introduced, the IFNγ levels in CD4^+^T cells, which were suppressed by mMDSCs, exhibited a significant elevation (**Figure 2C**). Furthermore, there was a tendency towards an increase in IFNγ levels from CD8^+^T cells suppressed by mMDSCs, although statistical significance was not reached (**Figure 2C**). These findings strongly suggested that DSP-0509 enhanced the anti-tumor immune activity of myeloid cells, including both TAMs and MDSCs.

### DSP-0509 reduced Treg function and differentiation, and when combined with IDO1 inhibitor, enhanced anti-tumor activity

**Figure 3:**
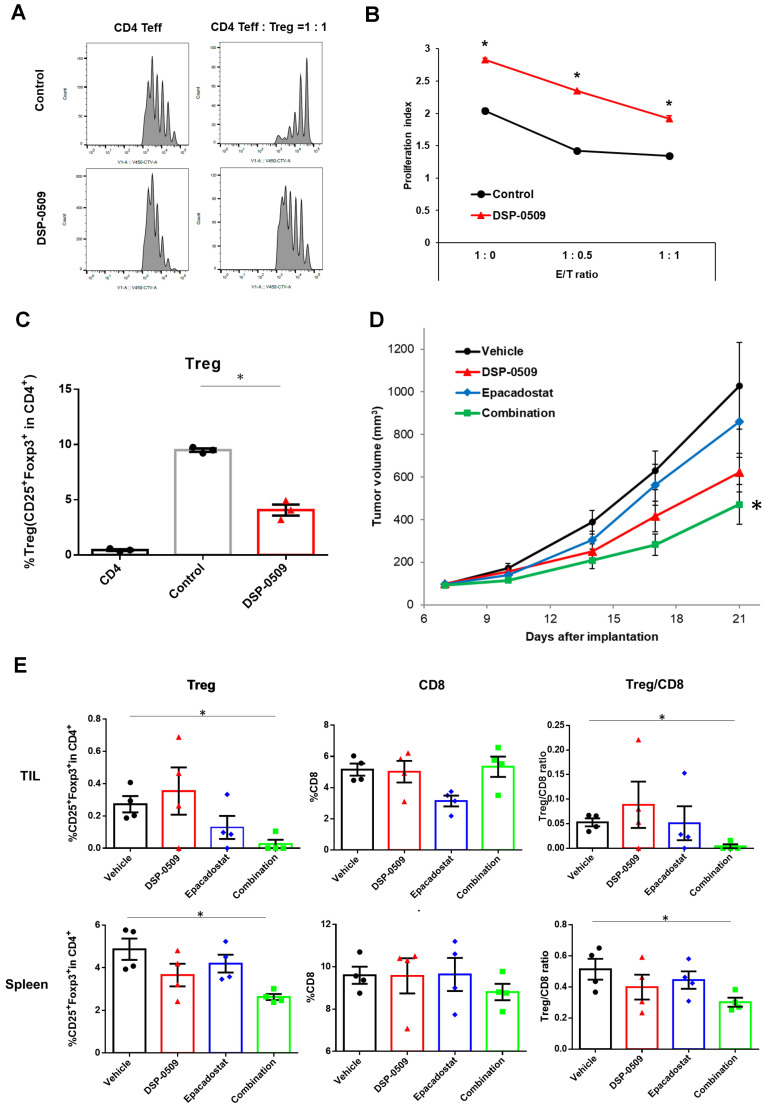
DSP-0509 reduced Treg function and differentiation, and when combined with IDO1 inhibitor, enhanced anti-tumor activity. **(A)** Representative histogram of CellTrace™ Violet (CTV) fluorescence. CD4 Teff cells were co-cultured with Tregs for 3 days. CD4 Teff cell proliferation was evaluated by the ratio of CTV fluorescence intensity. **(B)** The proliferation index was calculated based on CTV fluorescence intensity. *P < 0.01 two-tailed Student *t* test vs control. Values are the mean ± S.E.M. of each group, n = 3/group. **(C)** Tregs were differentiated from CD4^+^ cells in the presence of TGFβ and CD11c^+^ DCs. Tregs were detected as CD4^+^CD25^+^Foxp3^+^cells. *P < 0.05 for the τ-test. **(D)** Curve of HM-1 tumor growth. DSP-0509 5 mg/kg i.v. was administered once weekly and Epacadostat 100 mg/kg p.o. was administered once daily. *P < 0.05 for the Dunnett test. n = 6/group and the values are the mean and S.E.M. **(E)** TILs were isolated from HM-1 tumor at day 21. Tregs and CD8^+^ cells were analyzed by flow cytometry. *P < 0.05 for the Wilcoxon rank sum test.

Tregs exert immune suppression on anti-tumor responses by competing with effector T cells for costimulatory molecules on antigen-presenting cells and by secreting immunosuppressive molecules such as IL-10 and adenosine (22). Interestingly, previous reports have indicated that TLR7 agonists can inhibit the function and differentiation of Tregs (23, 24). However, these findings are inconsistent with our scRNA-seq results, which demonstrated an increase in Tregs within CT26 tumors following DSP-0509 treatment (**Figure 1C**). To validate the effects of DSP-0509 on Tregs, we isolated splenic Tregs and CD4^+^ T cells from CT26 tumors in tumor-bearing mice and subjected them to co-culture in order to assess the growth of CD4^+^ T cells (**Figures 3A, B**). Our observations revealed that CD4^+^ T cell proliferation was stimulated by the anti-CD3 antibody, but this was reduced when co-cultured with Tregs, suggesting that Tregs suppress the proliferation of CD4^+^ T cells (**Figure 3A**). However, the addition of DSP-0509 to the CD4^+^ T cell-Treg co-culture increased the proliferative fraction of CD4^+^ T cells (**Figure 3A**). Furthermore, DSP-0509 consistently enhanced the proliferation of CD4^+^ T cells independent of the CD4^+^ T cell:Treg ratio (**Figure 3B**). These results strongly indicated that DSP-0509 counteracts the inhibitory effects of Tregs on effector T cell proliferation. Next, to investigate the impact of DSP-0509 on Treg differentiation from CD4^+^ T cells, we co-cultured CD4^+^ T cells with CD11c^+^ DCs in the presence of TGFβ. In the control group, approximately 10% of CD4^+^ T cells differentiated into Tregs. However, the addition of DSP-0509 during the culture significantly reduced the percentage of Treg differentiation from CD4^+^ T cells (**Figure 3C**). These data confirm that DSP-0509 hampered Treg differentiation from CD4^+^ T cells. Despite its inhibitory effects on Tregs *in vitro*, DSP-0509 leads to an increased number of Tregs within the tumor microenvironment (**Figure 1C**). This discrepancy may be attributed to the influences of other immune cells, stromal cells, and cancer cells within the tumor microenvironment, or possibly because DSP-0509 specifically suppresses Treg function and generation without affecting their proliferation. Since IDO1 inhibitors have been proven to reduce Tregs within the tumor microenvironment (25, 26), we explored the potential of combining DSP-0509 with an IDO1 inhibitor to enhance the anti-tumor activity of DSP-0509 while simultaneously reducing Tregs. In the HM-1 tumor-bearing mouse model, the combination of DSP-0509 (5 mg/kg i.v. once weekly) and Epacadostat (100 mg/kg p.o. once daily) exhibited enhanced anti-tumor activity (**Figure 3D**). Furthermore, flow cytometry analysis demonstrated a significant decrease in the Treg fraction and Treg/CD8 ratios in both the tumor and spleen in the combination group (**Figure 3E**). MDSC including Monocytic MDSC (mMDSC) and PMN-MDSC in tumor were not altered in each treatment group (**Supplementary Figure 1**). These findings emphasized that DSP-0509 augmented anti-tumor immune responses by combining immunotherapy to suppress Tregs in the tumor microenvironment, while the effects of DSP-0509 alone primarily targeted Treg function and differentiation in the *in vitro* setting.

### DSP-0509 combined with anti-PD-1 modulated the tumor microenvironment and increased the size of the effector-like CD8^+^T cell cluster population

**Figure 4:**
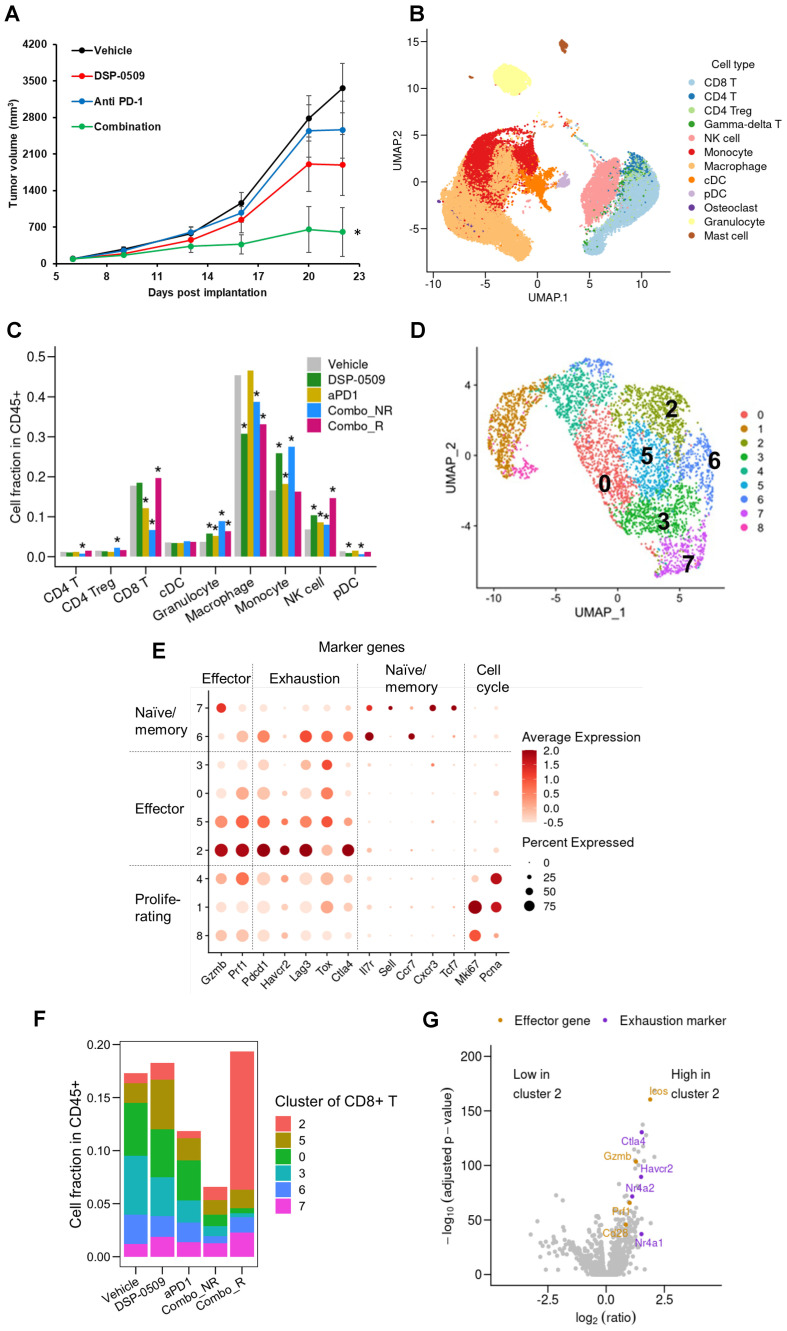
DSP-0509 combined with anti-PD-1 modulated the tumor microenvironment and increased the size of the effector-like CD8^+^T cell cluster population. **(A)** Tumor growth in CT26 model. DSP-0509 5 mg/kg i.v. was administered once weekly and anti-PD-1 antibody 200 μg was administered biweekly. *P < 0.05 for the Dunnett test. n = 6/group and the values are the mean and S.E.M. **(B)** UMAP representation of cell clusters in a CD45^+^ cell population derived from CT26 tumors. **(C)** Frequencies of identified immune cell populations in TILs. ^★^Adjusted P < 0.05 in the Fisher test. R, responder; NR, non-responder for combo therapy. **(D)** UMAP representation of cell clusters in a CD8^+^T cell population. **(E)** Expression profiles of marker genes for T cell subtypes in identified CD8^+^T cell clusters. **(F)** Frequencies of CD8^+^T cell clusters in each treatment group. **(G)** Comparison of gene expression between cells in cluster 2 and cells in other clusters shown as a volcano plot.

To assess the effect of a DSP-0509-anti-PD-1 antibody combination on immune cells, we administered DSP-0509 intravenously at a dosage of 5 mg/kg once weekly, in conjunction with an anti-PD-1 antibody intraperitoneally at a dosage of 200 μg/mouse twice weekly, to CT26 tumor-bearing mice. Notably, the combination treatment led to a remarkable suppression of tumor growth when compared to the individual agents alone (**Figure 4A**). In order to comprehensively investigate changes in immune cells within the tumor microenvironment, we conducted scRNA-seq on CD45^+^ TILs isolated from CT26 tumors across five experimental groups: control, DSP-0509 monotherapy, anti-PD-1 antibody monotherapy, responder to the combination treatment (Combo_R), and non-responder to the combination treatment (Combo_NR) (**Supplementary Figure 2**). The tumor samples used for scRNA-seq analysis were obtained 20 days after initiating the treatment regimen. A gene expression analysis of CD45^+^ cells successfully granted us access to approximately 3,000 gene expression profiles per cell. Employing clustering techniques on the gene expression data from all experimental groups revealed distinct subpopulations corresponding to different types of immune cells (**Figure 4B**). By scrutinizing the gene expression profiles of each cluster, we identified various immune cell types, including CD8^+^ T cells, CD4^+^ T cells, Tregs, NK cells, monocytes, macrophages, conventional dendritic cells (cDCs), pDCs, and granulocytes (**Figure 4B**). Next, we examined the distribution of immune cell percentages within each treatment group (**Figure 4C**). Notably, the DSP-0509 monotherapy group exhibited a significant increase in NK cells, monocytes, and granulocytes compared to the vehicle group. Conversely, macrophages and pDCs were significantly reduced. The anti-PD-1 antibody monotherapy group displayed a significant increase in NK cells, monocytes, and granulocytes, with a concomitant decrease in CD8^+^ T cells. In the combination responder group (Combo_R), the percentages of CD8^+^ T cells, NK cells, and granulocytes were significantly elevated, while macrophages were significantly reduced compared to the vehicle group (**Figure 4C**). Despite the modest increase in CD8^+^ T cell frequency, we postulated that the phenotype of CD8^+^ T cells might have undergone alterations, as the tumor growth inhibition was potentiated in the combination treatment group. To test this hypothesis, we isolated and separated the CD8^+^ T cell fractions into nine distinct clusters based on gene expression (**Figure 4D**). By assessing the expression of genes characteristic of well-known CD8^+^ T cell subtypes, we classified the cluster populations as effector T cells, memory/naïve T cells, and proliferating T cells (**Figure 4E**). Comparisons of cluster cell frequencies among the experimental groups revealed a considerable increase in cluster 2 cells within the combination responder group when contrasted with the other groups (**Figure 4F**). To gain insights into the phenotypic attributes of cluster 2, we identified genes differentially expressed in cluster 2 cells as compared to cells in the remaining clusters, demonstrated in the form of a volcano plot (**Figure 4G**). Notably, cluster 2 exhibited elevated expression of a set of genes associated with the activation of CD8^+^ T cells, including Icos, Ifng, Gzmb, and Cd28, in comparison to the other clusters (**Figure 4G**). Consequently, our findings suggest that the heightened antitumor effects observed with the combination of DSP-0509 and anti-PD-1, in relation to individual agents, are mediated through the activation of CD8^+^ T cells rather than an increase in CD8^+^ T cell infiltration into the tumor.

### DSP-0509 combined anti-PD-1 increased the level of M1-like macrophages in the tumor microenvironment

**Figure 5:**
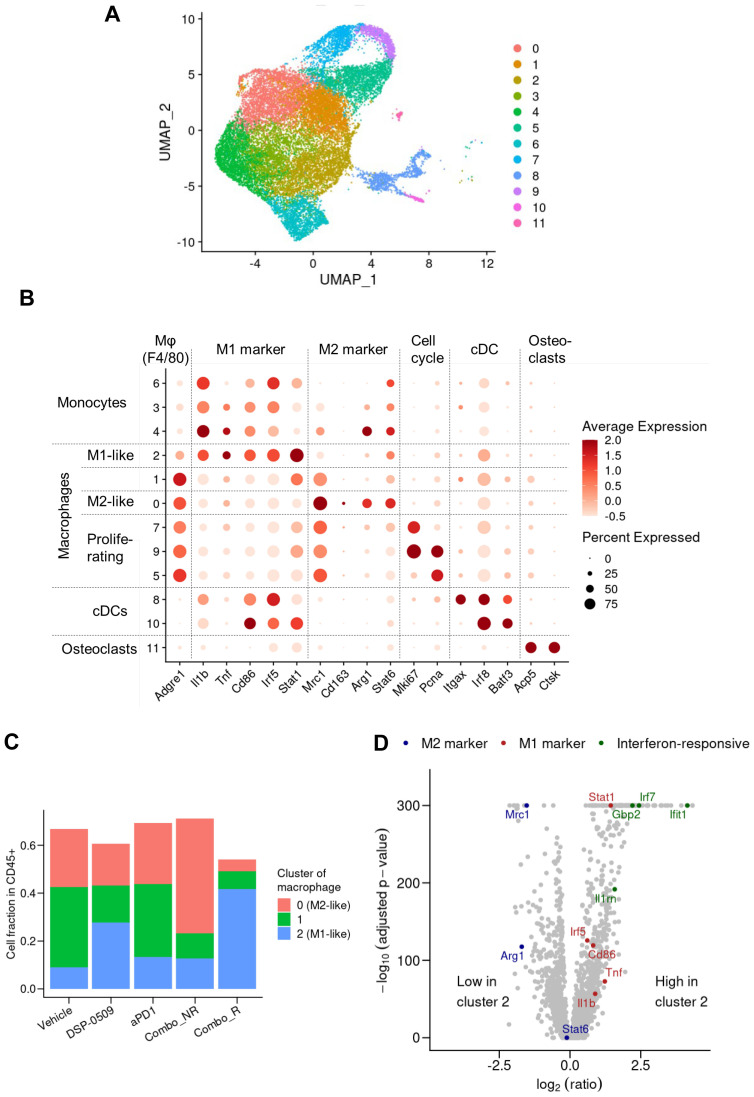
DSP-0509 combined anti-PD-1 increased the level of M1-like macrophages in the tumor microenvironment. **(A)** UMAP representation of monocyte, macrophage and cDC clusters in a population of CD45^+^ cells derived from CT26 tumors. Data in UMAP includes of all treatment group. **(B)** Expression profiles of marker genes for myeloid subtypes in macrophage clusters. Data in UMAP includes all treatment group. **(C)** Frequency of macrophages identified by cluster gating in each treatment group. **(D)** Comparison of gene expression in cluster 2 cells with that in the cells of other clusters shown by a volcano plot.

TLR7, a target of DSP-0509, is recognized to be considerably expressed in macrophages and is responsible for regulating macrophage function (27). Consequently, we performed an additional analysis with a specific focus on the macrophage fraction in the scRNA-seq data. The segregation of myeloid cells based on gene expression revealed 12 distinct subgroups encompassing DCs, monocytes, and macrophages (**Figure 5A**). Activation markers associated with macrophages such as Tnf, Cd86, and Stat1 displayed substantial expression in the cluster 2 population, indicating their M1-like macrophage phenotype (**Figure 5B**). Moreover, the augmented expression of Mrc1, Arg1, and Stat6 in the cluster 0 population suggested their M2-like macrophage phenotype (**Figure 5B**). Notably, in clusters 5, 7, and 9, the cells expressing both the growth marker Mli67 (Ki-67) and Pcna exhibited heightened expression of Mrc1 (CD206), signifying the presence of proliferating M2-like macrophages (**Figure 5B**). Interestingly, the gene expression profile of cluster 1 cells exhibited an intermediate phenotype between cluster 2 and 0, implying that TAMs are phenotypically diverse (**Figure 5C**). Comparison with other groups revealed a substantial increase in the fraction of M1-like macrophages and a significant decrease in the fraction of M2-like macrophages in the combination responder group (**Figure 5C**). Further examination of gene expression patterns between cluster 2 cells and other clusters revealed an upregulation of IFN-responsive genes and genes associated with M1 macrophages in cluster 2 (**Figure 5D**). These findings strongly suggested that DSP-0509, acting upon TLR7-expressing macrophages as a monotherapy, triggered anti-tumor immune responses within the tumor microenvironment. Furthermore, the combination of DSP-0509 and anti-PD-1 antibody profoundly drove the transition of macrophages from an M2-like phenotype to an M1-like phenotype.

### DSP-0509 combined with AXL inhibitor enhanced anti-tumor activity through additive upregulation of the anti-tumor phenotype of myeloid cells

**Figure 6:**
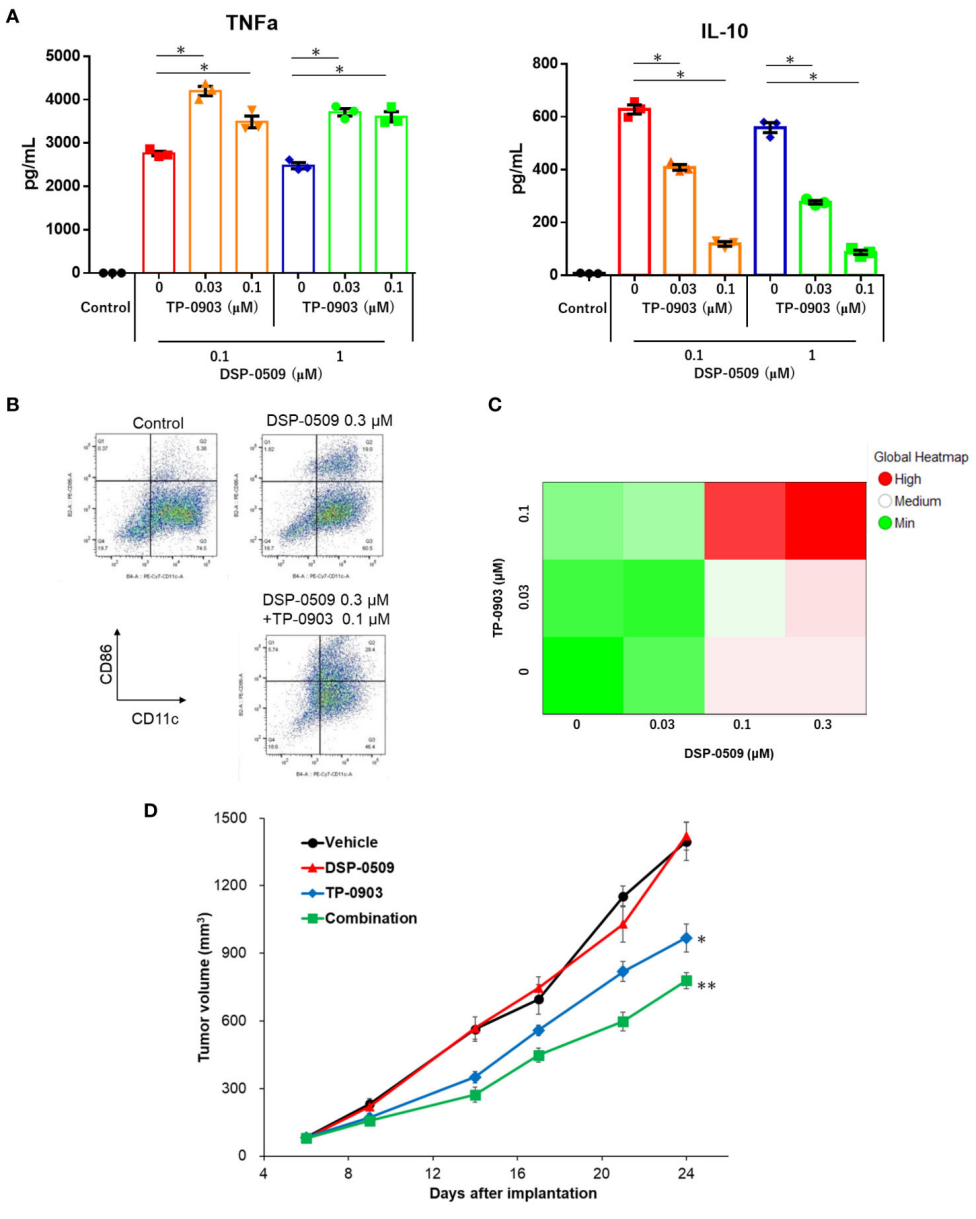
DSP-0509 combined with AXL inhibitor enhanced anti-tumor activity through additive upregulation of the anti-tumor phenotype of myeloid cells. **(A)** M2-like bone marrow derived macrophages (BMDMs) were induced to differentiate from bone marrow cells by the addition of M-CSF for 6 days. BMDMs were cultured in the presence of TP-0903 for 1 day, followed by DSP-0509 for 1 day before supernatant collection. *P < 0.01 in the parametric Dunnett test. **(B)** Representative dot plot of BMDCs after treatment with DSP-0509 alone or in combination with TP-0903. **(C)** BMDCs were cultured for 2 days in the presence of DSP-0509 alone or combined with TP-0903. Cells expressing CD86^+^CD11c^+^ (an activation marker of BMDCs) were analyzed. **(D)** Growth curve of tumors in the 4T1 orthotopic model. DSP-0509 1 mg/kg i.v. was administered once weekly and TP-0903 30 mg/kg was orally administered. **P < 0.0001, *P < 0.001 for the Dunnett test. n = 6/group and the values are the mean and S.E.M.

It has been reported that the activation of TLR7 in DCs leads to a reduction in TLR7 expression (28) and triggers the activation of compensatory signaling pathways (29). Consequently, the ability of TLR7 agonists to effectively activate cells within the myeloid system, thus exerting anti-tumor activity in the tumor microenvironment, may be compromised. Given that the inhibition of AXL has been shown to attenuate the activation of NF-kB by TLR signals (13, 14), it was hypothesized that inhibiting AXL would enhance the immune response against tumors when combined with DSP-0509. Thus, we sought to investigate whether the combination of DSP-0509, an AXL inhibitor, and TLR7 agonist induces robust activation of myeloid cells, resulting in a potent anti-tumor response within the tumor microenvironment. To assess the impact on immunosuppressive M2-like macrophages, which are abundant in tumors, bone marrow cells were incubated with M-CSF for 6 days to induce bone marrow-derived macrophages (BMDMs). Subsequently, TP-0903 was added to the BMDMs, followed by the addition of DSP-0509 after 1 day of culture. Analysis of IL-10 and TNFα concentrations in the supernatants demonstrated that treatment with DSP-0509 led to increased secretion of TNFα and IL-10 (**Figure 6A**). Conversely, the addition of TP-0903 significantly elevated TNFα secretion while markedly reducing IL-10 secretion (**Figure 6A**). Interestingly, elevated TNFα secretion was only observed when DSP-0509 was combined with TP-0903, not when administered alone (**Figure 6A**). These results suggested that AXL inhibition in macrophages inhibits the negative regulatory feedback of TLR7 signaling, leading to enhanced macrophage activation in comparison to treatment with DSP-0509 alone.

To further investigate the effect on DCs, bone marrow-derived dendritic cells (BMDCs) were generated by culturing bone marrow cells with GM-CSF for 9 days, followed by treatment with either DSP-0509, TP-0903, or a combination of both for an additional 2 days. Flow cytometry analysis of CD86 expression, a marker of DC activation (**Figure 6B**), revealed a dose-dependent increase in CD86 expression for each compound and their combinations (**Figure 6C**). Subsequently, we evaluated the anti-tumor activity of the DSP-0509 and TP-0903 combination in a 4T1 orthotopic mouse model by administering DSP-0509 (1 mg/kg, i.v., once weekly) together with TP-0903 (30 mg/kg, p.o.). Our findings demonstrated that TP-0903 at 30 mg/kg displayed a significant inhibitory effect on tumor growth, while DSP-0509 alone did not. Moreover, the combination of DSP-0509 and TP-0903 exhibited stronger tumor growth inhibition than TP-0903 alone (**Figure 6D**). These results suggested that AXL inhibitors enhanced the anti-tumor immune activity of DSP-0509 by promoting the activation of myeloid cells within the tumor microenvironment.

## Discussion

**Figure 7:**
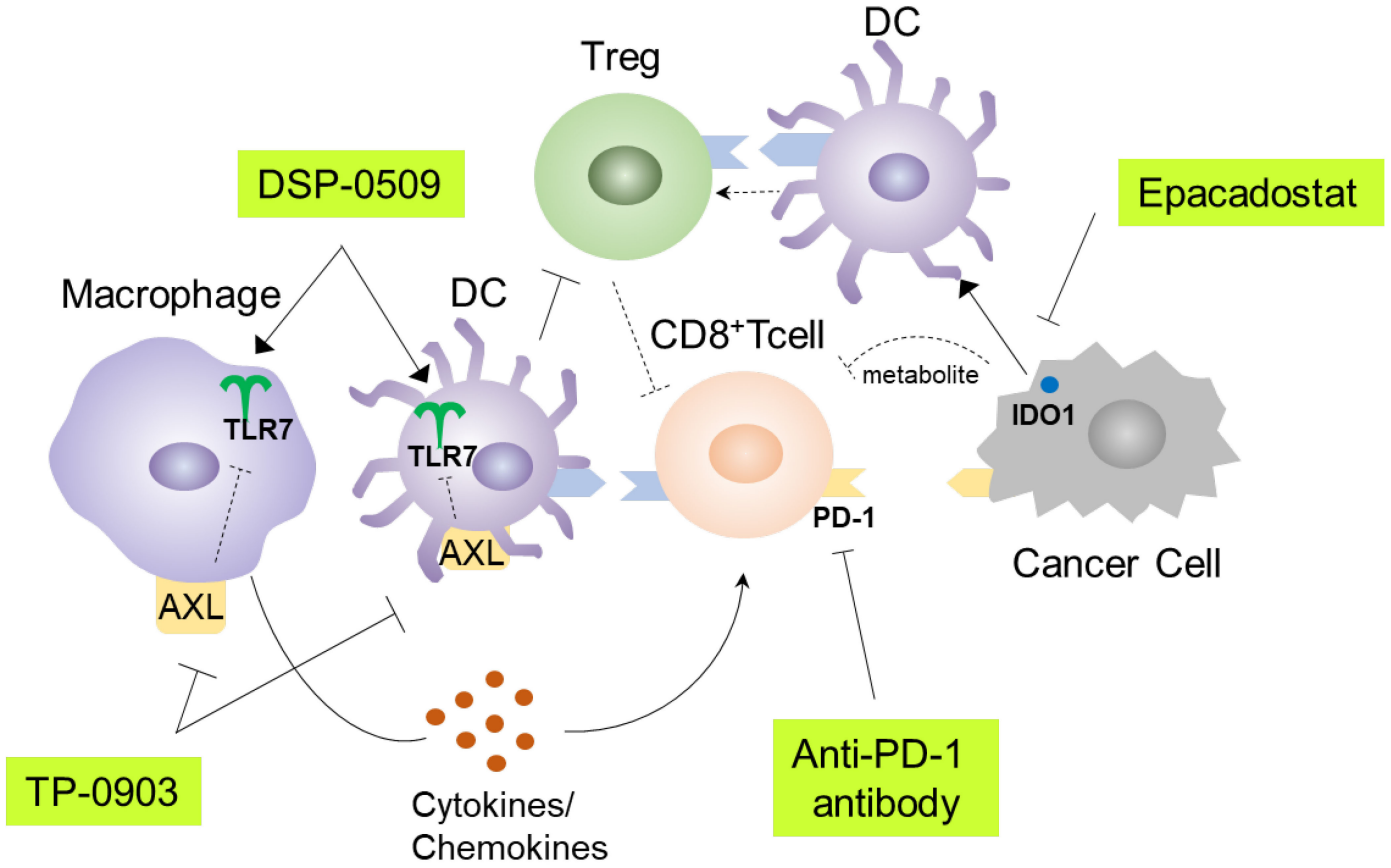
Graphical summary of this study.

TLR7 agonists have been shown to primarily target DCs and macrophages, leading to efficient uptake of antigens and presentation to cytotoxic T lymphocytes (10). Consequently, various immune cells in the tumor microenvironment are subsequently affected. These alterations can sometimes hinder anti-tumor immunity. To address this, combination immunotherapy may be a viable approach. DSP-0509 is designed to be rapidly excreted from the body and has a low risk of systemic immune adverse effects, including cytokine releasing syndrome. This can be partly explained by the fact that DSP-0509 is a substrate for the OATP transporter involved in its excretion (11). The TLR7 selectivity is one of the unique features of DSP-0509 compared to other TLR7 agonists, as most reported TLR7 agonists also exhibit TLR8 agonistic activity (30). The expression profile of TLR8 in immune cells is broader than that of TLR7, which may explain why TLR7 selective agonists have a wider safety margin (31). However, we should acknowledge the possibility that there may be differences between humans and mice regarding the response to TLR7 agonist, as the subset of DCs and the TLR7 expression profile in DCs are not always identical in both species (32). In our study, we demonstrated that DSP-0509 effectively modulated the function and infiltration of diverse immune cells within the tumor microenvironment. Furthermore, it exhibited compatibility with other immune-modulating agents, such as anti-PD-1 antibodies, AXL inhibitors, and IDO1 inhibitors (**Figure 7**). In the Treg suppression assay, DSP-0509 clearly mitigated the suppressive effect of Tregs on T cells, indicating its potential as an immunomodulator. Interestingly, scRNA-seq analysis revealed an increase in the proportion of Tregs infiltrating tumors following treatment with DSP-0509. Previous studies have reported variation in the ability of different TLR7 agonists to reduce Treg accumulation in different models (24), suggesting that Tregs may have a lesser impact in the CT26 model examined in our study. DSP-0509 also stimulated effector cells, including CD8^+^ T cells, leading to the upregulation of IFNγ expression. However, it is noteworthy that cancer cells, in response to IFNγ exposure, enhance immunosuppressive activity within the tumor microenvironment by upregulating the expression of immunosuppressive factors such as IDO1 (33). Therefore, we hypothesize that the reduction in Treg levels was more significant in tumors than in the spleens of mice treated with the combination of DSP-0509 and an IDO1 inhibitor.

We have demonstrated the ability of DSP-0509 to mitigate the immunosuppressive function of MDSCs. Our findings align with previous studies illustrating the inhibitory effect of TLR7 agonists on the immunosuppressive properties of MDSCs (34, 35). The therapeutic targeting of AXL has gained significant attention due to its prevalent overexpression in numerous cancer cells, as well as its involvement in the polarization of macrophages towards the M2-like phenotype (12). TP-0903 has been identified as a specific inhibitor of TAM family members, including AXL and MER (36, 37). AXL expression in macrophages and BMDCs has previously been reported to be upregulated by TLR activation (28, 38). Furthermore, the expression of AXL in macrophages is induced by type I interferon (38). Given that TLR7 stimulation strongly induces type I interferon expression in pDCs (39), it is plausible to consider AXL as a potent negative feedback regulator of TLR-mediated signals. In our study on BMDMs, pre-treatment with an AXL inhibitor augmented the secretion of TNFα induced by TLR7 stimulation while IL-10 secretion. These results suggested that prior administration of an AXL inhibitor, preceding DSP-0509 treatment, may further enhance the anti-tumor efficacy.

Previously, we reported the synergistic effect of combining DSP-0509 with an anti-PD-1 antibody, which resulted in the generation of memory CD8^+^T cells (11). In the present analysis using scRNA-seq, we observed an increased abundance of memory CD8^+^T cells, particularly in the combination responder group. Notably, clusters 6 and 7 were associated with memory CD8^+^T cells. Existing literature categorizes memory T cells into two subtypes, namely effector memory T cells and central memory T cells (40). In our study, cluster 6 cells were considered a fraction of effector memory T cells due to their high expression of Gzmb and relatively lower expression of CD127, a marker for central memory T cells. Intriguingly, our study revealed the subdivision of effector T cells into four distinct fractions, indicating their heterogeneous nature. Therefore, our present analysis may have captured the transient states of these cells. When examining the macrophage fractions in the group treated with the combination of DSP-0509 and anti-PD-1 antibody, we identified more than one distinct subset. Macrophages display M1-like differentiation through the NF-kB pathway in response to ligand binding to TLR7. However, it has also been reported that PD-L1 expression is concurrently upregulated (41). As increased PD-L1 expression in macrophages contributes to the exhaustion of CTLs, the simultaneous inhibition of the PD-1 signal in macrophages by the anti-PD-1 antibody may have synergistically enhanced the anti-tumor activity of the DSP-0509 and anti-PD-1 antibody combination. Furthermore, macrophage activity is hindered by PD-1 expression (41), suggesting that macrophage activity may play a crucial role in the anti-tumor mechanism of this combination therapy. Recent studies have highlighted the oversimplification of TAMs classification into M1 and M2 types. Instead, an increasing body of evidence suggests a more diverse array of macrophage phenotypes (42). Consistent with this notion, our scRNA-seq analysis identified various distinct fractions. By focusing on the gene expression profiles within each fraction, we further discovered clusters representing intermediate phenotypes of M1 and M2 TAMs, exemplified by cluster 1 (**Figure 4B**). Interestingly, the addition of the anti-PD-1 antibody to the combination therapy resulted in a reduction in the abundance of cluster 1 cells. This finding implies that the DSP-0509 and anti-PD-1 antibody combination may induce the differentiation of intermediate phenotype cluster 1 cells into M1-like cluster 2 cells. Recent studies have proposed the inclusion of tissue resident macrophages (TRMs) and monocyte-derived macrophages (MDMs) within the TAMs category. The classification of TRMs and MDMs poses a challenge due to variations in marker expression across different organs in humans (42). To classify the TRMs and MDMs is challenging because the marker of TRMs is different in each organ in human (42). To accurately determine the macrophage phenotype, it would be necessary to employ an orthotopic tumor model or a spontaneous tumor model.

In this study, we employed a state-of-the-art scRNA-seq methodology to investigate the precise alterations in immune cells within the tumor microenvironment following treatment with DSP-0509 or the DSP-0509-anti-PD-1 antibody combination. Furthermore, we demonstrated that the anti-tumor efficacy was markedly enhanced by co-administration of DSP-0509 with either an IDO1 inhibitor or an AXL inhibitor, although involvement of diverse immune cell except for Tregs and macrophages needs to be examined in the future study. These significant findings hold great promise for advancing the development of novel combination immunotherapies.

## Data Availability

The datasets presented in this study can be found in online repositories. The names of the repository/repositories and accession number(s) can be found below: https://www.ncbi.nlm.nih.gov/, PRJNA1091729 https://www.ncbi.nlm.nih.gov/, PRJNA1091370.
