# Supplementary material for: DSP-0509, a TLR7 agonist, exerted synergistic anti-tumor immunity combined with various immune therapies through modulating diverse immune cells in cancer microenvironment
Source: Front Oncol. 2024 Sep 13;14:1410373. doi: 10.3389/fonc.2024.1410373 (PMC11427241; doi:10.3389/fonc.2024.1410373)
Supplement: Supplementary file 1 [file DataSheet1.docx]

Supplementary Material

# Supplementary Figures and Tables

## Supplementary Figure 1.


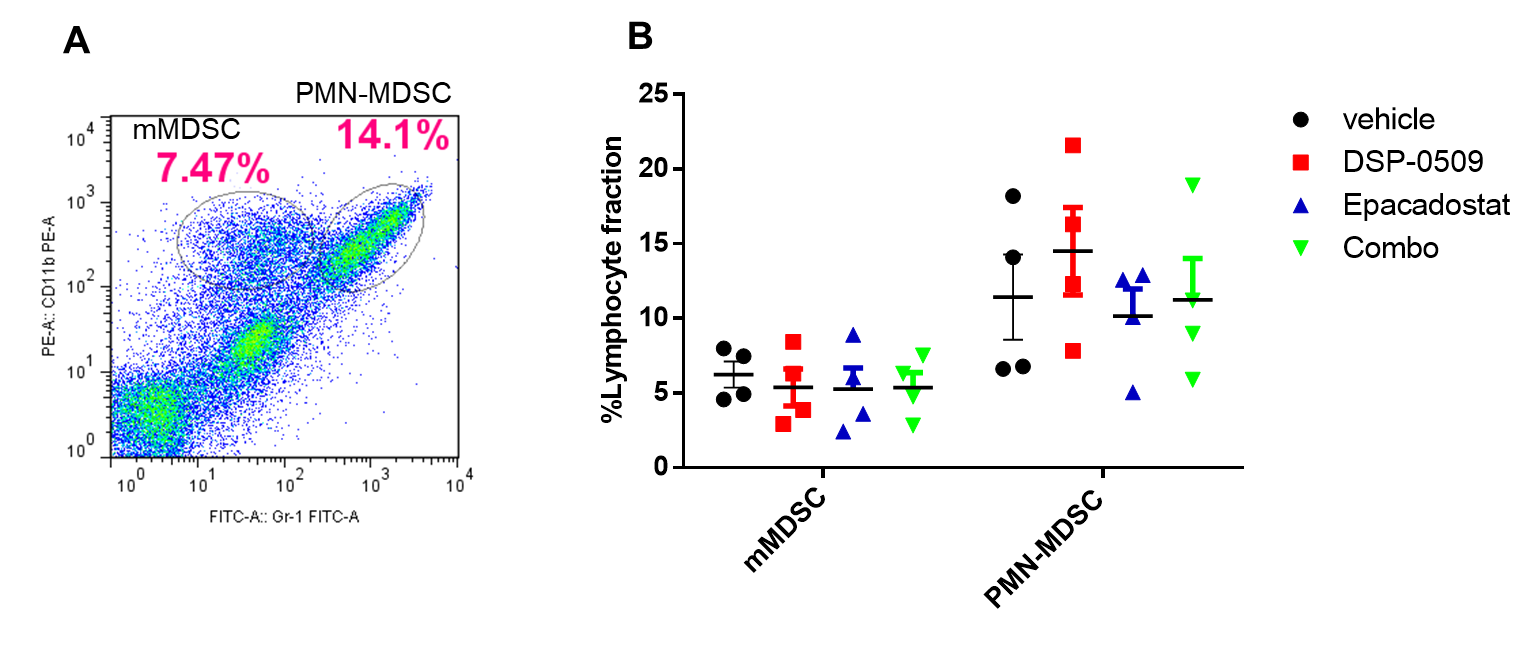


**Supplementary Figure 1.** MDSC infiltration in HM-1 tumor analyzed by flow cytometry. HM-1 tumors treated with each treatment were collected at day21. **A**. Representative histogram of MDSCs. Monocytic MDSC (mMDSC) and PMN-MDSC were defined as the CD11b^+^Gr-1^mid^ and CD11b^+^Gr-1^high^ respectively. **B**. The ratio of MDSCs in TILs in HM-1 tumor. n= 4 / group.

## Supplementary Figure 2


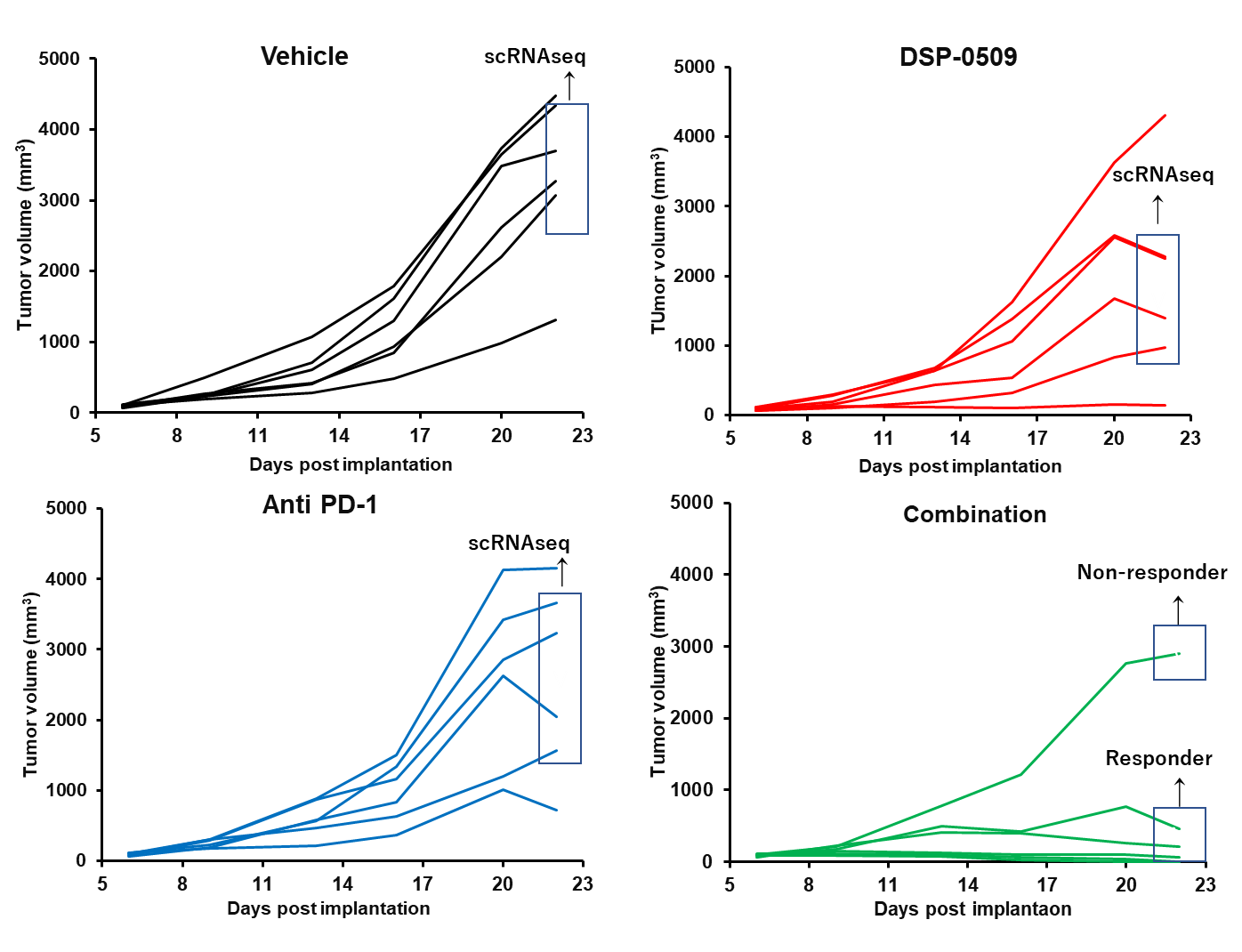


**Supplementary Figure 2.** Individual tumor growth in Fig. 3A. In vehicle, DSP-0509 and anti PD-1 group, TILs derived from 4 mice as indicated in figure above were pooled and used for scRNAseq. In combination group, TILs derived from 3 mice as indicated in figure above were pooled and used as responder. TILs derived from 1 mouse as indicated in figure above were used as non-responder.
